# Fabrication of cell plastics composed only of unicellular green alga *Chlamydomonas reinhardtii* as a raw material

**DOI:** 10.1007/s00253-022-12000-2

**Published:** 2022-06-09

**Authors:** Akihito Nakanishi, Kohei Iritani, Akane Tsuruta, Naotaka Yamamoto, Marina Watanabe, Nanami Ozawa, Masahiko Watanabe, Kuan Zhang, Ayaka Tokudome

**Affiliations:** 1 Graduate School of Bionics, Tokyo University of Technology, 1404-1 Katakuramachi, Hachioji, Tokyo, 192-0982 Japan; 2 School of Bioscience and Biotechnology, Tokyo University of Technology, 1404-1 Katakuramachi, Hachioji, Tokyo, 192-0982 Japan; 3 School of Engineering, Tokyo University of Technology, Hachioji, Japan

**Keywords:** Biomass plastics, Cell plastics, Carbon recycling, Green algae, *Chlamydomonas reinhardtii*, Intracellular contents

## Abstract

**Abstract:**

Cell plastics in this study were fabricated with only unicellular green alga *Chlamydomonas reinhardtii* as raw materials. The sizes of cell-major axis as structures were 8.4 ± 1.2 µm, and the aspect ratios of those were 1.2 ± 0.1, showing homogeneous particle size. After optimizing extraction condition of intracellular contents, cell plastics were fabricated with the cells as ingredient components and the intracellular contents as matrix components. Those cell plastics were observed with scanning electron microscopy, displaying the smooth surfaces of the cell plastics at a low magnification level. However, the surface, especially exposed surface, were rough at high magnification level. Tensile strength test revealed that increasing the ratio of intracellular contents in the cell plastics until 21% led enhancing mechanical properties of Young’s modulus and tensile strength; however, 25% of intracellular contents displayed decreases of those properties. As the optimal point, the cell plastic (21%), which contained 21% (*w*/*w*) of intracellular contents in cell plastics, showed 764 ± 100 MPa and 8.6 ± 5.2 MPa of Young’s modulus and tensile strength. The cell plastics showed few plastic region and soon fractured, indicating the possibility that cells and intracellular contents could be electrostatically connected. Additionally, cells were shown as a negative charge and displayed the possibility to contribute electrically cell-gathering with intracellular ionic components. Therefore, cells and intracellular contents containing ionic metabolites could be electrostatically connected for giving the mechanical strength to cell plastics. In this study, we successfully demonstrated fabricating cell plastics with only cells for the first time and also showed the high possibility of conjugating each cell with the intracellular contents.

**Key points:**

• *Cell plastics are fabricated with unicellular green algal cell directly.*

• *Unicellular cells required to be conjugated for the fabrication with matrix.*

• *Cells were conjugated with intracellular contents for cell-plastic fabrication.*

## Introduction

A plenty of various plastics (e.g., low-density polyethylene (LDPE) (Sabetzadeh et al. 2015); high-density polyethylene (HDPE) (Kiszka and Łomozik 2013), polypropylene (PP) (Bedia et al. 2000), polyvinyl chloride (PVC) (Chiellini et al. 2013), polystyrene (PS) (Schellenberg and Leder 2006), polyethylene terephthalate (PET) (Çaykara et al. 2020) were used on each field so those are essential materials to maintain modern grovel societies. Therefore, the request for sustainable and stable plastic-supplying is expected in the society, resulting that the recyclable raw materials of plastics will be considerably required (Nakanishi et al. 2020a). However, 99% of plastics are derived from non-recyclable petroleum as underground resources yet (Ciel 2017) and the amount of accumulation productions of plastics all over the world crossed over 8.3 Gt at 2015 and will be increasing toward future (Geyer et al. 2017), indicating that the plastic production will keep to be depended on the petroleum. In terms of the disposal of plastics, a plenty of plastics were abandoned and accumulated in environments. According to several trial calculation in scientific reports, 4% of plastic wastes were overflowed via rain and river and caused considerably bad effects (Green et al. 2015; Jain and Tiwari 2015; Mastafa et al. 2018; Pathak et al. 2014); 1% of the Pacific Ocean (1.6 million km^2^/165 million km^2^) was covered with micro plastics due to the influx of the plastic waste into the oceans (Lebreton et al. 2018). Then, to dissolve those problems of the production and the disposal of the petroleum-derived plastics, the researches and developments (R&D) of the biodegradable bioplastics not relied on the petroleum have been vigorously progressed all over the world (Nakanishi et al. 2020a). Recently, the cell plastics produced of unicellular green alga were reported as the bioplastics having the responsibility (Iritani et al. 2021; Nakanishi et al. 2020a, 2020b, 2020c, 2021a; Nakanishi and Iritani 2021). The main reason why the bioplastics have not become common so far is that the processes of extracting and refining the raw materials are time- and money-consuming; however, the cell plastics directly use cells as a raw material and could avoid those processes, expectedly resulting of cost cut. Additionally, the cell plastics are also expected to be as biodegradable materials because of using universal green algal strains in environment. So far, green algae *Chlamydomonas reinhardtii* was mainly used as an ingredient of the cell plastics for the following reasons: a cell’s rigid structure with crystalline layer (Goodenough et al. 1986); 10 ~ 50 times higher carbon assimilation activity than most terrestrial plants (Wang et al. 2008); the availability of genome information (Merchant et al. 2007), metabolic pathways (Chapman et al. 2015), and genetic engineering tools (Tran and Kaldenhoff 2020); being less problematic in the biosafety (Murbach et al. 2018) and so on. Additionally, *C. reinhardtii* is a unicellular microorganism so the cells can be freely placed to fabricate the cell plastics. On the other hands, the unicellular microorganisms require matrix to connect each cell so that the previous cell plastics were fabricated with processed starches, polybutylene succinate, and so on as the matrix component. Since the additions of the matrix components from other origins are time- and money-consuming, the no matrix preparation from others is expected to progress the system fabricating the cell plastics.

In this study, to evaluate cell properties for fabricating cell plastics, size frequency distribution. and aspect ratio of *C. reinhardtii* cells were firstly evaluated as particle materials. Secondly, to prepare intracellular contents as matrix components, cell-disruption method was optimized with ultrasonic homogenization. Thirdly, to fabricate the cell-plastics smoothly, the method of molding cell plastics was evaluated especially for drying process. For the fabrication, the cell plastics were made by mixing cells with containing different ratio of the intracellular contents. Those cell plastics were analyzed and evaluated regarding the surface structure of cell plastics by scanning electron microscopy (SEM), water repellency, and mechanical characteristics by test of tensile strength (Young’s modulus, maximum stress, and breaking elongation). Additionally, the reason why cells conjugated each other on cell-plastic fabrication was considered with experimental data. This is a first paper to demonstrate the fabrication of the cell plastics with only green algal cells and to evaluate the connection between cells and intracellular contents.

## Materials and methods

### Preparation of microalgal strain and evaluation of growth and cell structure

#### *Chlamydomonas reinhardtii* strain C-9

NIES-2235 was cultured under the photobioreactor (PBR) equipped 200 µmol photons·m^−2^·s^−1^ based on a previous our study (Nakanishi et al. 2021b). The cultivation medium was Modified Bold’s basal medium (MBBM): 1.5 mM NaNO_3_, 0.22 mM K_2_HPO_4_, 0.3 mM MgSO_4_·7H_2_O, 0.17 mM CaCl_2_·2H_2_O, 0.43 mM KH_2_PO_4_, 0.43 mM NaCl, and necessary components described in a previous report (Berges et al. 2001). The cell growth was evaluated as dry cell weight (DCW) using a value of optical density (OD) of 750 nm with a spectro-photometer U-2900 (Hitachi, Tokyo, Japan) via appropriate calibration curve for OD_750_ versus DCW as before-nitrogen-depletion version. Nitrate concentration was evaluated with an appropriate calibration curve for absorbance of 220 nm (Abs_220_) versus nitrate concentration after obtaining supernatant of the broth (Nakanishi et al. 2021b). Cell sizes were measured, and cells were taken pictures with an optical microscope CKX53 (Olympus, Tokyo, Japan).

### Evaluation of cell viability

The cell viability was evaluated by a staining method with neutral red: 180 µL of broth was mixed with 20 µL of a solution of neutral red (red pigment) (Tokyo Kasei Co., Ltd., Tokyo, Japan) for 5 min (Crippen and Perrier 1974). The neutral red solution was prepared as below: neutral red was overdosed in 1 mL of phosphate-buffered saline (PBS) (137 mM NaCl; 8.1 mM Na_2_HPO_4_; 2.7 mM KCl; 1.5 mM KH_2_PO_4_, pH = 7.4); the solution was filtered with 0.22 μm filter (Nylon Syringe Filter, Membrane-Solutions). The cells after staining were washed with PBS, and the stain-treated cells were evaluated as living cells on cell counter plates (Fukae Kasei Co., Ltd., Kobe, Japan).

### Cell disruption of *C. reinhardtii*

Ultrasonic homogenization was conducted as below: an ultrasonic homogenizer Smurt NR-50 M operating at 20 kHz with an ultrasonic horn and a 3-mm-diameter tip NS-50 M-MT3 (Funabashi, Chiba, Japan) was employed. The harvested cells were suspended with 1 mL of PBS in 2 mL polypropylene centrifuge tube. After the preparation, 1 cm of the end of the chip was inserted into the cell suspension, and the cell suspension was sonicated (on: 30 s; off: 30 s, 5 cycles) on ice.

### Quantification of extracted chlorophyll a/b

Chlorophylls were collected from the cells cultivated at 92 h with ultrasonic homogenization. After evaluating cell density in the broth using a OD_750_-DCW calibration curve, 1 ~ 20 mg of cells was harvested by centrifugation at 5000 × *g* for 3 min at 23ºC and the supernatant was discarded. The collected cells were treated by cell-disruption method shown as above. To measure the quantities of chlorophyll a/b, data of Abs_665_ and Abs_652_ of the collected solution were obtained after subtracting Abs_750_ for a baseline correction using PBS with a spectrophotometer U-2900 (Hitachi, Tokyo, Japan) and substituted in the formula as below, chlorophyll a (µg·mL^−1^): 16.72 × Abs_665_ – 9.16 × Abs_652_; chlorophyll b (µg·mL^−1^): 34.09 × Abs_652_ – 15.28 × Abs_665_ (Nassour et al. 2017). The contents of chlorophyll a/b in the cells were finally shown using the unit µg·mg-DCW^−1^. After disruption, the cells were observed with an optical microscope CKX53 (Olympus).

### Preparation of cell plastics

Broth containing 150 mg-DCW of cells was centrifuged at 5000 × *g* for 3 min at 23ºC, and supernatant was discarded. Collected cells were mixed with intracellular contents derived from 10, 20, and 30 mg of cells as matrix components and loaded onto mold for the next drying treatment. Two patterns of drying treatment were performed at 80 °C for 24 h as heat treatment and at 23 °C for 24 h as non-heat treatment, respectively.

### Observation with scanning electron microscopy

The cell plastics were coated with Au particles using an ion coater (IB-2; Eiko Engineering, Tokyo, Japan) before scanning. The coated cell plastics were observed with SEM (JSM-6060LV; Japan Electron Optics Laboratory Co., Ltd., Tokyo, Japan).

### Water repellent evaluation test

Water repellency of each cell plastic was observed after depositing a drop of water on the cell plastic with Drop Master 300 (Kyowa Interface Science Co., Ltd., Saitama, Japan).

### Evaluation of Young’s modulus, maximum stress, and breaking elongation

The Young’s modulus and tensile strengths of the cell plastics were evaluated with a tensile strength tester (TesTex, Zurich, Switzerland) (Iritani et al. 2021; Ma et al. 2015; Nakanishi et al. 2020b, 2020c, 2021a). The cell plastics were cut into rectangles. The maintained cross-head rate was 1.00 mm·min^−1^. A load–displacement curve was plotted under analyzing the test samples. To calculate Young’s modulus, maximum stress and breaking elongation, a stress–strain (s–s) curve was plotted by dividing the load and displacement by the cross-section and the initial length of the test samples, respectively. The thickness of the cell plastics was measured by the SEM observations towards the cross-sections of the test samples. Young’s modulus was obtained with the slope of an initial straight line approximated via the least-squares method. The maximum stress was defined as the maximum value of stress on tensile strength. The Young’s modulus, maximum stress, and breaking elongation of each film were the averages of three test samples obtained by cutting three different films.

### Analysis of extracellular electric charge

Cells corresponding to 3 mg-DCW in broth were collected by centrifuged at 5000 × *g* for 1 min at 23ºC. The cells were directly loaded into well of 1.5% of agarose gel. Electrophoresis was performed with 100 V in tris–Acetate-EDTA (TAE) buffer. The cell precipitation was demonstrated with cells corresponding to 3 mg-DCW in PBS containing 0 ~ 100 mg of aluminium sulfate.

## Results

### Cell preparation

**Fig. 1:**
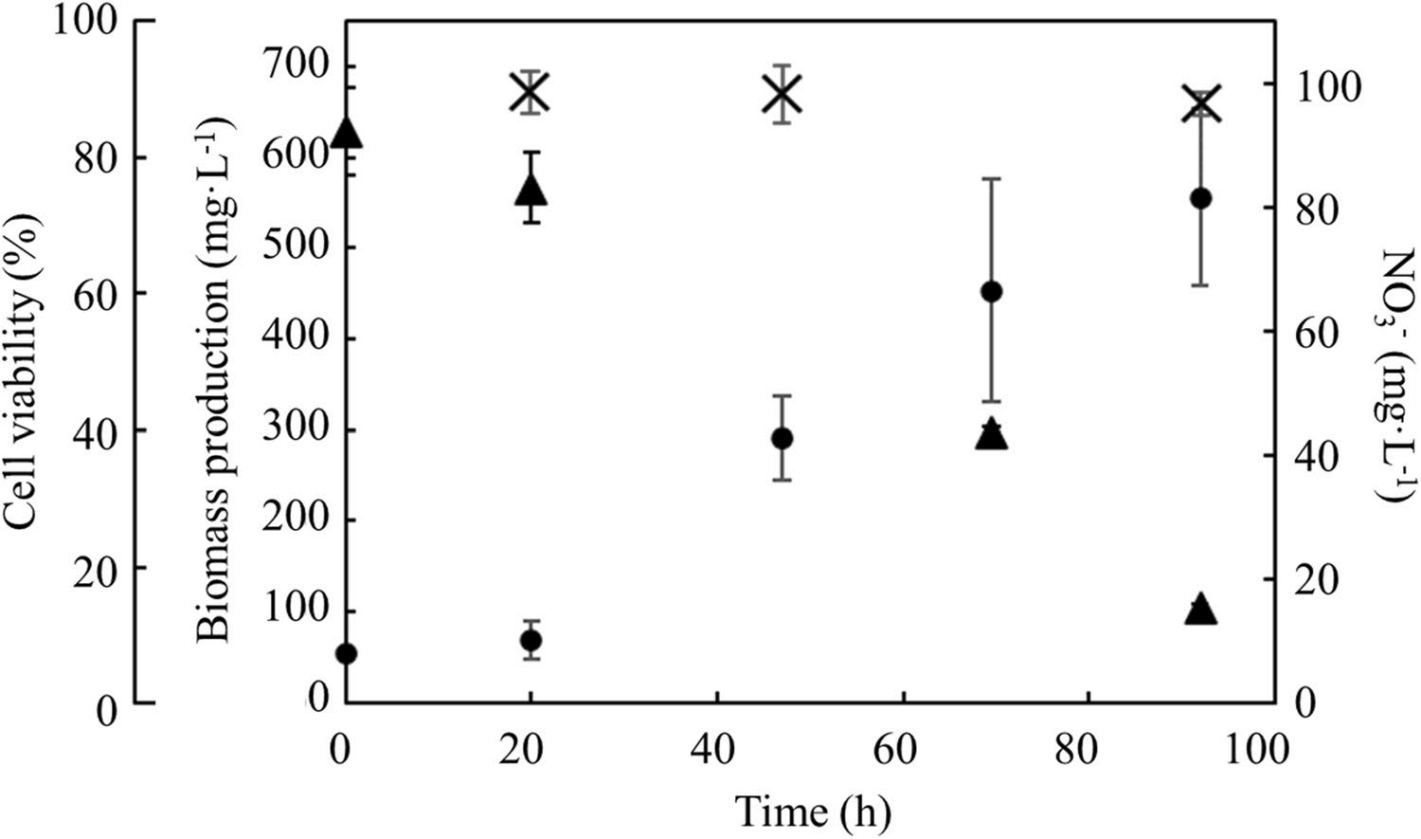
Time course profiling of growth. Time course profiling of growth of *C. reinhardtii* was represented by biomass production (●), nitrate concentration (▲), and cell viability ( ×). Values are the averages of three replicated experiments, ± SD

**Fig. 2:**
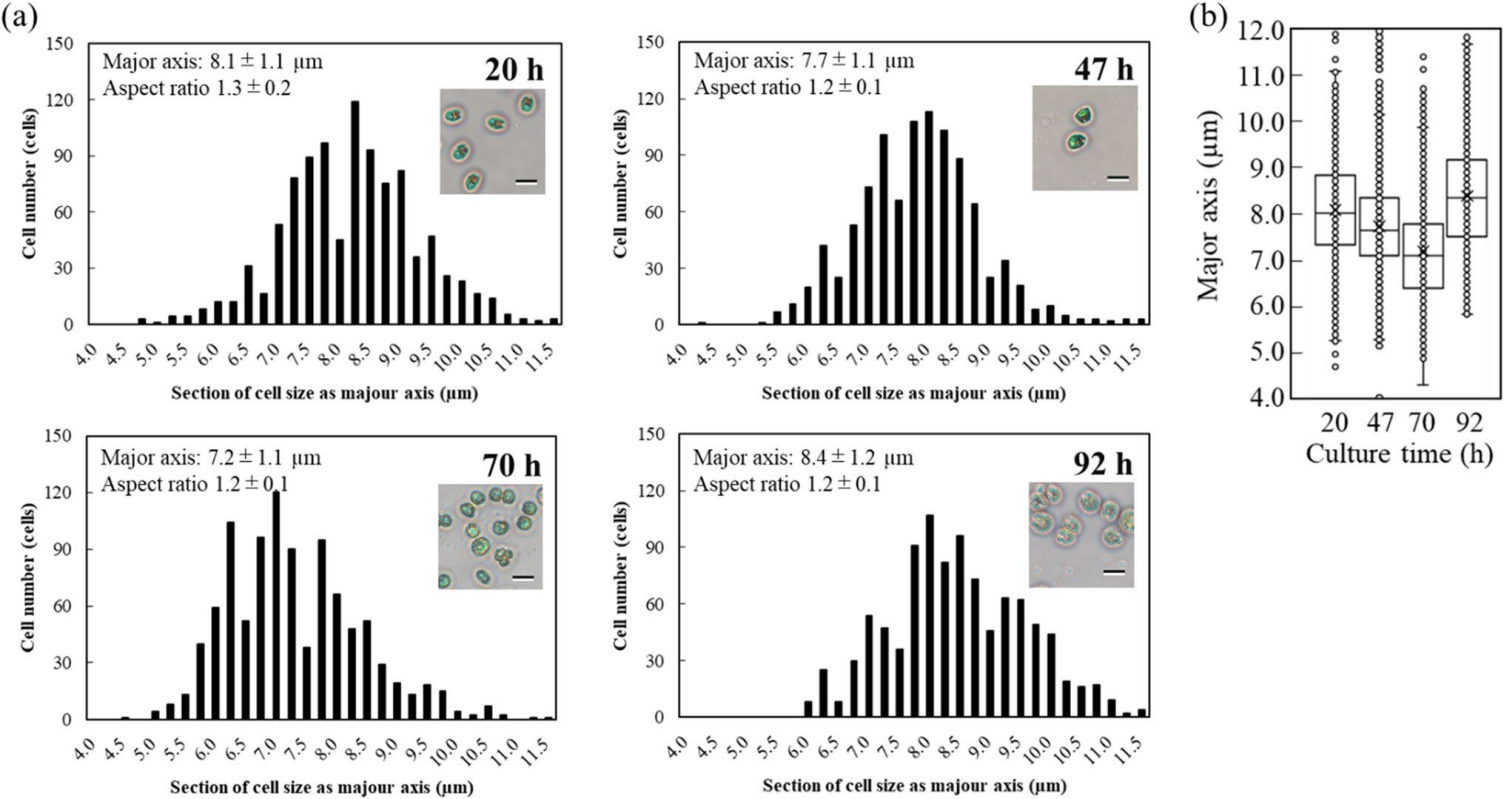
Sizes of cells of *C. reinhardtii* strain C-9: NIES-2235 at each culture time. Cell size distribution and aspect ratio were shown. **a** Histogram: sizes of cells of *C. reinhardtii* strain C-9: NIES-2235 at each culture time were measured under optical microscopy (scale bar: 10 µm, *n* = 1000, respectively). Sections of the size as major axis were divided every 0.5. Aspect ratios were shown as a value as major axis/minor axis of each cell. **b** Box plot: length of major axis was plotted at 20 h, 47 h, 70 h, and 92 h of culture time

To evaluate growth condition of supplied cells, DCW of *C. reinhardtii* and nitrate concentration in PBR were analyzed (Fig. 1). The biomass production and productivity were 556 mg·L^−1^ and 145 mg·L^−1^·d^−1^ at 92 h before nitrogen depletion. In this study, the cell viability at 92 h was 88.0 ± 1.7%. The cell shape at each culture time was investigated to evaluate cell’s outer structure as for using cell-plastic resource (Fig. 2). As the results, the sizes of *C. reinhardtii* C-9: NIES-2235 in this study were 8.1 ± 1.1 µm, 7.7 ± 1.1 µm, 7.2 ± 1.1 µm, 8.4 ± 1.2 µm at 20 h, 47 h, 70 h, and 92 h, and those data could not deny null hypothesis (Fig. 2a). The distributions of major axis size of cells were measured with box plots in detail (Fig. 2b). The results showed that the medians and the boxes, in which the 50% of diameter-data were plotted, behaved similarly. Although the medians gradually decreased 8.2 µm, 7.7 µm, and 7.2 µm at 20 h, 47 h, and 70 h, the median increased 8.3 µm at 92 h. Those aspect ratios were 1.3 ± 0.2, 1.2 ± 0.1, 1.2 ± 0.1, and 1.2 ± 0.1 at 20 h, 47 h, 70 h, and 92 h, indicating that the cells at each culture time were almost spherical shapes without significant differences (Fig. 2a).

### Extraction of intracellular contents

**Fig. 3:**
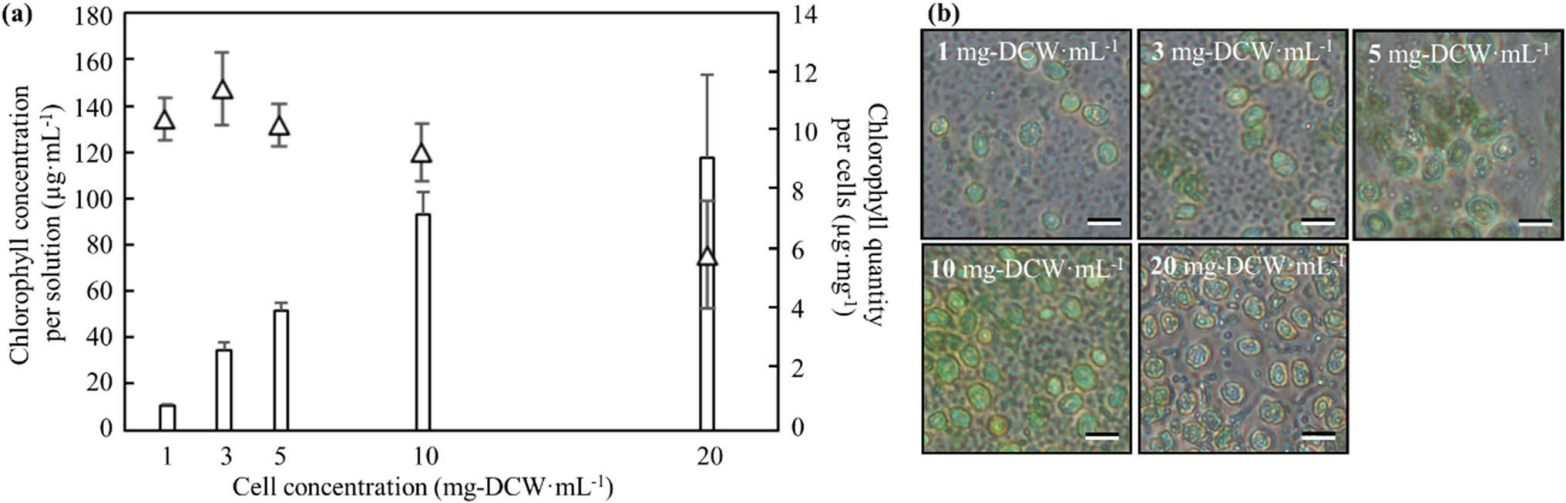
Evaluation for the extraction of intracellular contents by using ultrasonic homogenization. Intracellular contents were extracted from cells cultivated at 92 h by ultrasonic homogenization. **a** Chlorophyll concentrations per solution and chlorophyll quantity per cells were represented by bars and triangles. Values are the averages of three replicated experiments, ± SD; **b** cells treated by ultrasonic homogenization (1, 3, 5, 10, and 20 mg-DCW·mL.^−1^) were observed by optical microscope with 10 µm scale bar

In this study, the cell plastics were fabricated by filling intracellular contents among cells as matrix components. To optimize efficiency of collection of intracellular contents, the extraction condition with ultrasonic homogenizer was evaluated by using data of quantities of chlorophyll (a + b) extracted from cells (Fig. 3a). With 1 mL of PBS as extraction solution, 9.3 ~ 11.5 µg·mg-DCW^−1^ of chlorophyll (a + b) was collected from 1 ~ 10 mg of cells. On the other hands, 5.9 µg·mg-DCW^−1^ of chlorophyll (a + b) was collected from 20 mg of cells, meaning that the extraction efficiency was significantly depressed. Then, 10 mg-DCW·mL^−1^ was set as the optimized extraction condition. Observing the cells with an optical microscope after the sonication, several cells that were not perfectly crushed (Fig. 3b).

### Properties of fabricated cell plastics

**Fig. 4:**
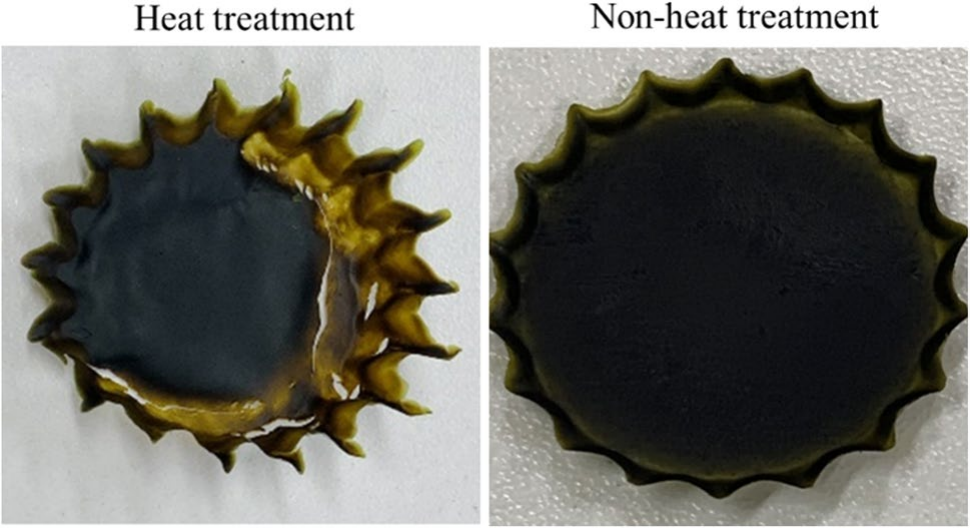
Pictures of cell plastics under different drying processes. Cell plastics mixing 150 mg-DCW of cells with intracellular contents derived from 20 mg of cells were prepared w/or w/o heat treatment, respectively

**Fig. 5:**
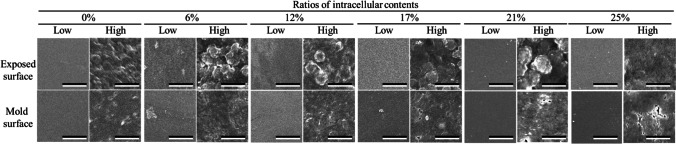
SEM images of cell plastics. Cell plastics with intracellular contents (0% ~ 25%) were prepared without heat treatment. Scale bars were 500 μm and 10 μm in low (× 20) and high (× 1000) magnification levels, respectively

**Fig. 6:**
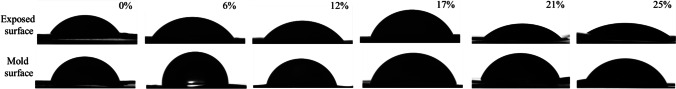
Evaluation of water repellency of each cell plastic. Cell plastics with intracellular contents (0% ~ 25%) were prepared without heat treatment, respectively. Water repellency on exposed and mold surfaces were represented in upper row and lower rows

**Fig. 7:**
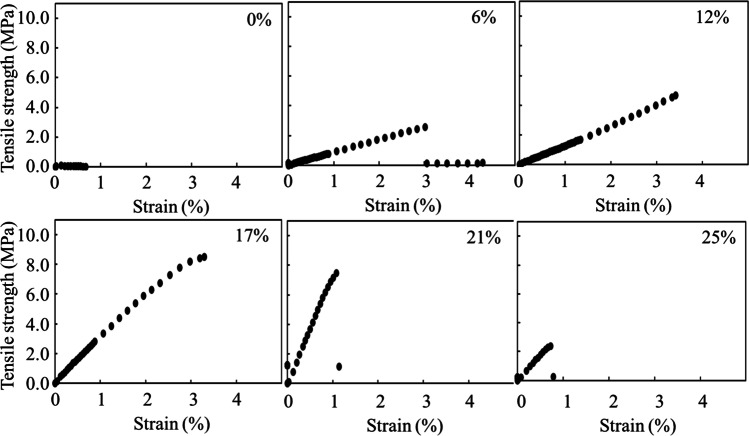
Analysis of s–s curves for each cell plastic. Cell plastics contained with 0% ~ 25% of intracellular contents were fabricated without heat treatment. The data from test of tensile strength were plotted as s–s curve

**Fig. 8:**
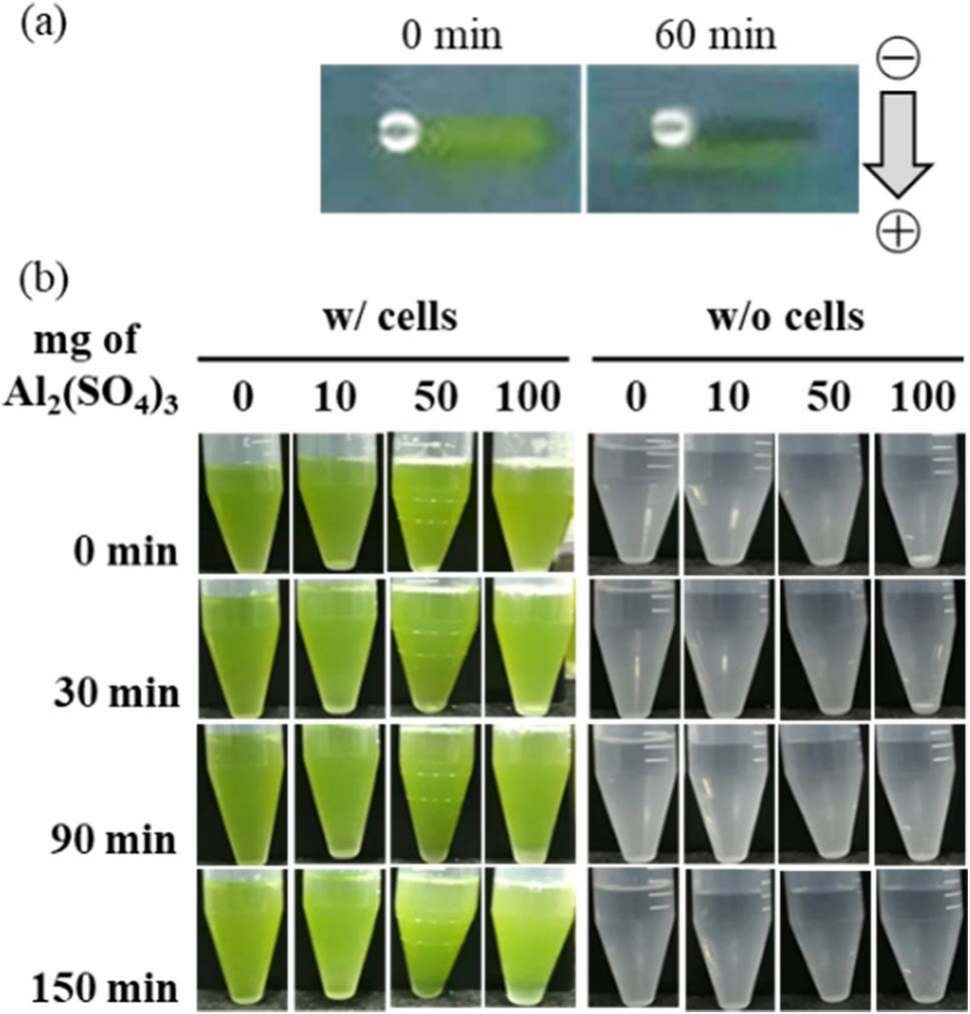
Analysis of extracellular electric charge. Extracellular electric charge of cells was evaluated: **a** cells were directly loaded into well in agarose gel and electrophoresis was performed in TAE buffer with 100 V; **b** cell precipitation was demonstrated with aluminium sulfate

The cell plastics were fabricated with cells and intracellular contents as only sources from *C. reinhardtii*. The mixture of the cells and the intracellular contents was molded under each dry condition at 23 °C (room temperature) or 80 °C (Fig. 4). The cell plastics dried at 23 °C were nearly uniform thickness and plane surface; on the other hands, those dried at 80 °C were the structure biased of cell existence. The condition at 23 °C was suitable for the fabrication of trimmed cell plastics so that the cell plastics prepared by air-drying method were evaluated. The six types of cell plastics were named as cell plastics (0%), cell plastics (6%), cell plastics (12%), cell plastics (17%), cell plastics (21%), and cell plastics (25%), meaning that those cell plastics contained 0%, 6%, 12%, 17%, 21%, and 25% of the intracellular weights versus the total cell plastic weight. To evaluate fabricating the cell plastics with the cells as structures, the surfaces of cell plastics were observed with SEM (Fig. 5). As the results, at law magnification, the surfaces of all cell plastics were smooth without rough deposits. On the other hands, at high magnification, the structure-like cells were observed on the exposed surfaces (exposed surfaces) of the cell plastics compared to mold surfaces (surfaces in contact with mold). Water repellency is an important item in the several properties of plastics. Herein, the water repellency of each cell plastic was evaluated (Fig. 6). According to the criterion of water repellency (Rosado and Holder 2013), less than 40° of contact angle means that the plastic is superhydrophilic. All the cell plastics in this study demonstrated over 40° of contact angle on mold surface so that those showed water repellency not as superhydrophobic. The water repellency was well shown on mold surface compared to exposed surface. In order to evaluate the strain resistance and toughness of the cell plastics, s–s curve of each cell plastic was drawn (Fig. 7). The s–s curves revealed that all the cell plastics showed few plastic region and soon fractured, meaning of hard and fragile mechanical properties. According to more detailed analyses of the s–s curves, increasing the intracellular contents until 21% led enhance Young’s modulus from 6 ± 5 MPa to 764 ± 100 MPa, and similarly maximum stress from 0.5 ± 0.5 MPa to 8.6 ± 5.2 MPa; however, the contents to 25% showed decreasing Young’s modulus (371 ± 20 MPa) and maximum stress (4.6 ± 3.7 MPa) (Table 1). The properties of the cell plastics could be compared to ones of polybutylene succinate and LDPE since polybutylene succinate exhibits a Young’s modulus of 240 ± 90 MPa and a tensile strength of 8.8 ± 3.8 MPa and LDPE also displayed Young’s modulus of 165.3 MPa and a tensile strength of 10.3 MPa (Brandrup et al. 1999; Nakanishi et al. 2020c). On the other hands, although the intracellular contents until 17% also showed enhanced breaking elongation from 0.7 to 3.0 ~ 4.0%, the contents to 21% and 25% displayed decrease of the breaking elongation to 0.8 ~ 1.2%. The properties of the cell plastics could be compared to ones of polybutylene succinate and LDPE since polybutylene succinate exhibits a Young’s modulus of 240 ± 90 MPa and a tensile strength of 8.8 ± 3.8 MPa [14] and LDPE also displayed Young’s modulus of 165.3 MPa and a tensile strength of 10.3 MPa [18]. Only intracellular contents could not be independent as a film so the mechanical property of the intracellular contents was not evaluated (data not shown). The electrical charge of cells was evaluated to show the possibility of ionic cell conjugation with intracellular contents (Fig. 8). The result of electrophoresis with cells showed the cell migration in well of agarose gel to the anode side, indicating that the cells were negatively charged. Furthermore, the cells in PBS containing aluminum sulfate caused the precipitation depending on the concentration of aluminium sulfite.

**Table 1:** Correlation between Young’s modulus and intercellular contents for each cell-plastic. The cell-plastics prepared with intracellular contents were prepared without heat treatment, respectively. Values are the averages of three replicated experiments, ± SD.

Ratio of intracellular contents(%)	Young’s modulus (MPa)	Maximum stress (MPa)	Breaking elongation (%)
0	6 ± 5	0.5 ± 0.5	0.7 ± 0.0
6	66 ± 3	2.9 ± 1.2	4.0 ± 0.9
12	137 ± 7	4.9 ± 0.7	3.0 ± 0.6
17	263 ± 63	7.0 ± 3.0	3.4 ± 0.9
21	764 ± 100	8.6 ± 5.2	1.2 ± 0.5
25	371 ± 20	4.6 ± 3.7	0.8 ± 0.3

## Discussion

In our culture system, the biomass production and productivity were 556 mg·L^−1^ and 145 mg·L^−1^·d^−1^ at 92 h before nitrogen depletion (Fig. 1), indicating few stress growths because of few differences of previous studies (Banerjee et al. 2021). Additionally, the cell viability was 88.0 ± 1.7% at 92 h and similar to 82.9% at nitrogen depletion in previous data (Nakanishi et al. 2021a, 2021b). The poor growth condition could cause cell structure to be weaken or broken (Sathe and Durand 2015) and provoke degraded robustness as raw materials of cell plastics; therefore, the growth condition should be carefully evaluated to guarantee the structural strength as appropriate cell plastic materials. According to the data of major sizes and aspect ratios in Fig. 2, the cells of *C. reinhardtii* C-9: NIES-2235 showed approximately 7.2 ~ 8.3 µm of spherical structures even at each culture time. The reason why the cell size increased from 70 to 92 h might be that those cells prepared for cell division. In this study, the size of cells at 92 h using as the materials of cell plastics was 8.38 ± 1.18 µm. The diameter size of this strain in this study was bigger than the one of *C. reinhardtii* CC-125: 4 µm; on the other hands, the size was smaller than the one of *C. reinhardtii* CC-2931 and *C. reinhardtii* CC-2342: 10 µm (Sathe and Durand 2015). Following the meaning of standard deviation, 68.3% of cells cultured at 92 h maintained 7.2 ~ 9.6 µm so the prepared cells were particles with high uniformity.

According to the data in Fig. 3, the efficiency of extraction was decreased when the introduced amounts of the cells were increased to 20 mg-DCW·mL^−1^. The result was not suited at the point of view for the extraction process but guaranteed the cell robustness at the point of view for structure. As the next step, the cell plastics might be fabricated directly by using the cell mixture treated under the optimal ultrasonicating condition. Then, according to previous report regarding the extraction efficiency of chlorophyll with the sonication (Gerde et al. 2012), our efficiency was not poor. Although there is possibility not completely to be extracted intracellular contents in this study, the extraction efficiency could be superior to previous condition in another study. And also, the quantity of extracted chlorophyll at 10 mg-DCW·mL^−1^ with ultrasonication, 9.3 ± 1.0 µg·mg-DCW ^−1^, was almost similar to the quantity of extracted chlorophyll with methanol, 15.2 ± 3.8 µg·mg-DCW ^−1^ (Nakanishi et al. 2021b), indicating that the extracting efficiency with the sonication was few deference with the methanol extracting methods.

The cell plastics produced under different drying conditions were evaluated in Fig. 4. The evaluation revealed that controlling water in the raw material affected fabricating the cell plastics during the process. As the reason, at 80 °C drying, the mixture of cells and intracellular contents rapidly lost water, resulting in loss of fluidity, and the cells were biasedly placed. On the other hands, at 23 °C drying, the mixture gradually lost water, resulting in keeping fluidity, and the presence of cells was not biased.

According to the results of the SEM image in Fig. 5, cell-like structures were fewer on mold surface than on exposed surface. As the reason, during the drying process, cells could be lower density than intracellular contents and floated to exposed surfaces. Not expecting the lotus effect, the reason why the water repellency was better on mold surface than on exposed surface might be that hydrophobic intracellular contents were settled and accumulated on mold surface during molding (Fig. 6). In fact, the cells of *C. reinhardtii* almost in nitrogen depletion contain 10% (*w*/*w*) of lipids (Nakanishi et al. 2020b), possibly indicating the possibility of increasing water repellency depending on the accumulation of the component containing the lipids on mold surface.

The s–s curve of each cell plastic was drawn as mechanical properties (Fig. 7). The results meant that all cell plastics in this study were immediately ruptured when those caused the cracks rather than gradually broken after reaching the yield point. Those properties did not change with varying ratios of intracellular contents filled between cells, which might be related to the fact that varying ratios of the matrix contents showed also few improvements of breaking elongation. In previous our researches, the cell plastics were fabricated with polybutylene succinate and starch as matrix components (Nakanishi et al. 2020c, 2021a). Polybutylene succinate and starch could be as films not but powders, so those matrices showed the mechanical properties themselves indicated by the test of tensile strength, respectively. Therefore, following higher the ratios of polybutylene succinate and starch in the cell plastics, and the more the mechanical properties of each of these matrices were exhibited. In the other words, the mechanical properties of those cell plastics decreased as the ratios of cells in the cell plastics increased. The results could indicate that those matrices did not directly adhere to the cells but were simply mixed with cells. On the other hands, in this study, the cell plastics were fabricated with the intracellular contents as the matrix components. The intracellular contents were not films so the mechanical properties derived from the test of tensile strength could not be shown (data not shown). Then, even though the ratios of intracellular contents in the cell plastics increase, the mechanical properties of the cell plastics could not increase unless the intracellular contents directly adhered to the cells. Although Young’s modulus and maximum stress steadily increased from 0 to 21% of intracellular contents in the cell plastics, those properties decreased to 25% of those (Table 1). The cell plastics could be strengthened with the cells even if the intracellular contents were not self-independence as films; the Young’s modulus and maximum stress reduced even if the cell contents are excessive towards to the cells, suggested that the intracellular contents could successfully work to combine each cell.

As shown in Fig. 7, analysis of s–s curves for each cell plastic, the cell plastics showed few plastic region and soon fracture. The results suggested that cells and intracellular contents could be electrostatically connected. As the results in Fig. 8, the cell migration towards anode in the well by the electrophoresis and the precipitation of cells with aluminium sulfite showed that the electric charge of cell was negative and could contribute electrically cell-conjugating with ionic components. Therefore, cells and intracellular contents containing ionic metabolites could be electrostatically connected for giving the mechanical strength to cell plastics.

- In conclusion, cell plastics, containing intracellular contents as a matrix component, were successfully fabricated with only green alga *C. reinhardtii*. The cell plastics were simply fabricated with a mold at 23 °C as room temperature. The study towards the fabricated cell plastics revealed that the cells were composed as a structural material; the surface performed repellency; Young’s modulus and maximum stress were enhanced with an optimal ratio of intracellular contents versus cells. The electric charge of cells was negative so that cells could be electrically conjugated with intracellular contents. This is the first paper to demonstrate fabricating cell plastics with only cells and also to show the possibility of electrically conjugating each cell with the intracellular contents for the fabrication of cell plastics.

## Data Availability

All data generated or analyzed during this study are included in this article (and its supplementary information files).
